# Mobilising Implementation of i-PARIHS (Mi-PARIHS): development of a facilitation planning tool to accompany the Integrated Promoting Action on Research Implementation in Health Services framework

**DOI:** 10.1186/s43058-022-00379-y

**Published:** 2023-01-09

**Authors:** Sarah C. Hunter, Bo Kim, Alison L. Kitson

**Affiliations:** 1 Caring Futures Institute, Flinders University, Sturt Road, Bedford Park, South Australia 5042 Australia; 2 College of Nursing and Health Sciences, Flinders University, Sturt Road, Bedford Park, South Australia 5042 Australia; 3 Center for Healthcare Organization and Implementation Research, VA Boston Healthcare System, 150 South Huntington Avenue, Boston, MA 02130 USA; 4 Department of Psychiatry, Harvard Medical School, 25 Shattuck Street, Boston, MA 02115 USA

**Keywords:** Implementation science, Knowledge translation, i-PARIHS framework, Implementation tools, Facilitation

## Abstract

**Background:**

Facilitation makes the Integrated Promoting Action on Research Implementation in Health Services (i-PARIHS) framework a popular framework in the field of implementation science. Facilitation allows for flexible application of the i-PARIHS framework by encouraging the iterative tailoring of implementation strategies to a dynamic context. However, successfully harnessing this flexibility can be challenging to navigate, particularly for novice facilitators. Therefore, to support and promote more widespread use of the i-PARIHS framework, and to make it easier for people who are already using i-PARIHS, we have undertaken the Mi-PARIHS Project—Mobilising Implementation of i-PARIHS, focused on developing a suite of practical and pragmatic i-PARIHS resources.

**Methods:**

Through a co-design approach drawing on end-users’ experiences, we developed the Mi-PARIHS Facilitation Planning Tool, and this article reports on the final end-user feedback via an online survey.

**Results:**

A total of 58 participants completed the online survey. The survey focused on participants’ previous experiences with i-PARIHS, their feedback on the background information provided with the Mi-PARIHS Tool, and their feedback on the tool itself (e.g. clarity, use, satisfaction, improvements). This feedback resulted in the development of a comprehensive 34-item Mi-PARIHS Facilitation Planning Tool that supports i-PARIHS users in their (1) assessment of the i-PARIHS framework’s innovation, context, and recipient constructs; (2) development of a tailored facilitation plan; and (3) repeated use over time to evaluate the effectiveness of facilitation strategies.

**Conclusions:**

The Mi-PARIHS Facilitation Planning Tool makes framework-guided implementation more accessible and reliable to a wider range of systems and stakeholders, thereby contributing to more consistent implementation of evidence-based practices and other innovations. It addresses the challenge of systematically assessing core constructs of the i-PARIHS framework to develop tailored facilitation strategies. The Mi-PARIHS Facilitation Planning Tool is freely available for use at the website https://www.flinders.edu.au/caring-futures-institute/Mi-PARIHS-tool.

**Supplementary Information:**

The online version contains supplementary material available at 10.1186/s43058-022-00379-y.

Contributions to the literature

- The development of the Mi-PARIHS Facilitation Planning Tool in this paper provides i-PARIHS users with a systematic and theoretically informed way to plan their facilitation approach.
- This paper addresses a key contemporary issue in implementation science: balancing the need for being adaptive to context and complexity with the need for fidelity to evaluate implementation success (or failure).
- We have generated a novel approach to documenting and managing implementation and facilitation without undermining the significance of managing complexity and remaining flexible in our approaches.

## Background

High-quality research alone does not guarantee the uptake or adoption of research evidence or knowledge into practice [1]. There remains a consistent underuse, overuse, or misuse of research knowledge in clinical practice [2–5]. To address this, the field of implementation science provides systematic approaches, frameworks, and theories to inform the successful implementation of research into practice [1].

Early implementation research was not often theory-informed, with a review identifying only 10% of studies having an explicit rational or theory-informed approach to implementation [6]. This is despite the field of implementation science highlighting the importance and utility of theory-guided implementation [7]. Without an underpinning theory guiding implementation efforts, it is difficult to understand what elements influence implementation success or failure [8]. Furthermore, using theoretical approaches allows us to tease out why particular evidence is successfully implemented in one setting and not another [8].

Therefore, the past two decades have seen a significant increase in theory-informed implementation. Various researchers from a multitude of backgrounds have developed a multitude of theories, models, and frameworks to plan, guide, and evaluate implementation efforts, with recent research suggesting more than 100 approaches being used in implementation research [9]. A theory can be considered explanatory or predictive, a model is a simplified representation of a system, and a framework outlines the basic structure and components underlining a system [10].

One such framework, the Integrated Promoting Action on Research Implementation in Health Services (i-PARIHS), is a conceptual framework that aims to represent the dynamic interplay of factors that influence successful implementation [11]. i-PARIHS has an underlying philosophy that implementing research into practice is complex, unpredictable, and non-linear. Therefore, i-PARIHS was developed to support complex multi-disciplinary team-based interventions [12].

To support this complexity, i-PARIHS specifies core constructs (innovation, recipients, context, and facilitation) which influence successful implementation and is explicitly underpinned by relevant theories of innovation, behavioural and organisational change, and improvement [11]. Facilitation is positioned as the ‘core ingredient’ in relation to the other constructs and is specified as both a specific role (‘being’ a facilitator) and a set of actions (‘undertaking’ facilitation) [12]. Facilitation, as defined within i-PARIHS, is a set of actions or strategies that are interactive and context-responsive, which enable implementation and address barriers as they emerge in the implementation context [12].

Therefore, i-PARIHS argues that successful implementation results from the facilitation of an innovation with the intended recipients in their contextual setting. i-PARIHS represents an evolution of the PARIHS framework [13, 14], responding to several criticisms of that framework by providing clearer theoretical underpinnings as well as practical tools and case studies to help clinicians and researchers operationalise the framework [11, 12].

In addition to this revised theory and clearly specified framework elements, the developers of i-PARIHS provide several tools to operationalise i-PARIHS in practice, outlined in their facilitation guide [12]. This includes a clear description of facilitator attributes, skills, and roles, outlining the facilitator’s journey from a novice to an expert facilitator, a facilitation checklist to support a structured assessment of the framework constructs, and a facilitator’s toolkit to guide action. Furthermore, since the original publication of PARIHS in 1998, many researchers have developed tools to assist with the assessment of PARIHS and i-PARIHS constructs, for example, the Alberta Context Tool [15].

Despite the evolution and improvement of the PARIHS framework to the i-PARIHS framework, there continues to be feedback that the framework remains complex to apply in practice. Facilitation is what makes the i-PARIHS framework unique, and it makes the framework flexible in its application, by encouraging iterative tailoring to a dynamic context. However, harnessing this flexibility requires a nuanced understanding of the core constructs, as well as the complex potential roles and activities of a facilitator, which may be challenging for a novice facilitator to navigate [16].

A recent citation analysis of the original PARIHS framework identified 367 published articles that have used the framework in implementation research [17]. However, very few studies were identified to use the framework in a comprehensive way—e.g. have the framework guide earlier through later phases of an implementation effort. This citation analysis highlighted the popularity of this framework but also the significant need for providing resources to support the use of the framework more comprehensively. Furthermore, a recent case study identified that the resources included in the i-PARIHS Facilitation Guide [12] offer assistance [18]. Specifically, it was identified that the Facilitation Checklist and Facilitator’s Toolkit can be adapted for a range of projects and can be used within pre-implementation planning, implementation, and evaluation phases. However, it was noted that more explicit guidance and/or tools for using the content of the Facilitation Checklist and Facilitator’s Toolkit are required to help develop structured implementation plans and monitor fidelity to implementation plans and record how strategies are tailored to an evolving context.

To respond to feedback that more explicit guidance is required, to promote more informed use of the i-PARIHS framework, and to make it easier for people who are already using i-PARIHS, we have developed the ‘Mi-PARIHS Facilitation Planning Tool’, a comprehensive 34-item tool that (1) assesses the core constructs of innovation, context, and recipients; (2) generates a visual representation of barriers and enablers; (3) assists in developing a tailored facilitation plan; and (4) can be used at pre-implementation or can be used as a repeated measure over time to evaluate the effectiveness of facilitation strategies.

We have worked collaboratively with a range of end-users, aligning with the philosophy of the i-PARIHS framework, to develop a tool to support those conducting implementation research. Specifically, we want people to use the i-PARIHS framework in ways that ensure their implementation efforts are both theory-informed and pragmatic, enabling them to focus on and prioritise the theory-based i-PARIHS constructs over many other potential factors that could potentially impact implementation.

## Methods

This study is part of a broader project that seeks to adapt the i-PARIHS framework into a suite of practical and pragmatic resources (called the Mi-PARIHS Project—Mobilising Implementation of i-PARIHS). The current study reports on the results of a survey that collected end-user feedback on a previously developed Mi-PARIHS Facilitation Planning Tool. This tool was iteratively developed using a collaborative approach. Authentic collaboration involves engaging these users in all phases of the research process [19]. Therefore, development was a collaboration between the Mi-PARIHS team and various implementation researchers and clinicians who agreed to share their experience and feedback to inform the ongoing development and refinement of Mi-PARIHS resources. To bring together the end-users’ experiences in a collaborative way, the process to develop the Mi-PARIHS Facilitation Planning Tool was guided by the experience-based co-design toolkit [18]. This toolkit outlines 5 steps for co-design—(1) set up for success, (2) gather the experience, (3) understand the experience, (4) improve the experience, and (5) monitor and maintain the experience. Prior work was conducted to complete steps 1 through 3 [17, 18, 20, 21], and the current study reports on step 4. The ‘Discussion’ section outlines the steps we have set up for the fifth and final step. Before outlining the details and methods of step 4 conducted in this study, where we collected end-user feedback, we firstly provide some background detail of the previously completed steps 1 through 3 that informed the development of the tool.

### Method for steps 1–3 tool development

Step 1, *set up for success*, involves understanding what will contribute to success and then planning and investing effort into this. Since 2018, considerable time has been spent trying to understand what needs and demands there are for resources to support the use of i-PARIHS. The Mi-PARIHS team engaged with various stakeholders, including clinicians, health service managers, academics, researchers, and project managers, to understand their needs and experiences, and this ultimately informed the decision to create a tool via a co-design process.

Step 2, *gathering experience*, involves getting a sense of what the current experience is. In 2018, authors SCH and BK conducted a 1-h workshop at the North American Primary Care Research Group’s International Conference on Practice Facilitation, Tampa, FL. This workshop provided a useful opportunity to understand the needs and experiences of researchers, clinicians, and facilitators, and how useful they find the i-PARIHS framework and what they would want from any resources [20]. This workshop and the feedback collected from it informed the development of a preliminary tool.

Supplementing the information gathered at the workshop, a team of researchers, led by colleagues in Sweden, conducted a citation analysis of the original PARIHS framework. This review aimed to understand the breadth and depth of how the PARIHS framework has been used. This piece of work identified that much of the published use of PARIHS described a constrained use of the framework (e.g. used only for one phase of implementation) [17], demonstrating a need for greater support in how to use the framework more comprehensively.

Step 3, *understanding the experience*, involves taking the knowledge to stimulate further discussion and dialogue. Therefore, the Mi-PARIHS team collaborated with four implementation project teams who agreed to share their experience of using i-PARIHS and the tools they developed in order to operationalise the framework in an implementation project. This resulted in the development of in-depth case studies which provided useful insight into how users operationalise i-PARIHS and identified key areas for the development of support and resources [18].

The information gathered from the workshop, citation analysis, and case studies were used by the Mi-PARIHS team to develop a refined Mi-PARIHS Facilitation Planning Tool. Authors SCH and BK presented this tool to a group of end-users via the Implementation Facilitation Learning Collaborative in two 1-h interactive virtual sessions hosted by the Behavioural Health Quality Enhancement Research Initiative Program of the United States Department of Veterans Affairs [21]. The feedback from these sessions was fed back into the Mi-PARIHS Facilitation Planning Tool for refinement.

### Method for step 4 end-user feedback

Step 4, *improve the experience*, involves translating the understanding of the current experience into meaningful improvements. The information gained throughout all stages to date has been fed back into the tool. However, prior to publishing and releasing the Mi-PARIHS Facilitation Planning Tool for public use, we wanted to provide end-users with the opportunity to see the near-final version of the tool and provide survey-based feedback and comments. Therefore, the current study reports on the results of this survey.

To gain feedback from participants who understand the i-PARIHS framework and have prior experience in applying it in practice, we used purposeful sampling and targeted our recruitment to participants who were identified as all corresponding authors from the included studies in the PARIHS citation analysis [17] and all project team leads included in the i-PARIHS case study who are not already on the Mi-PARIHS team [18]. This included a total of 371 potential participants. All participants were emailed an invitation to review the Mi-PARIHS Facilitation Planning Tool and provide their feedback and comments via a Qualtrics survey. The survey, inclusive of reviewing the tool, was designed to take 20 min, and all responses were de-identified to keep participants’ feedback anonymous. The survey included some questions about participants’ demographic information, questions about participants’ previous experience and use of i-PARIHS, questions related to the information and introduction to the Mi-PARIHS Facilitation Planning Tool, and then specific questions related to the useability (e.g. is it user friendly, would they use it, how they would use it) and acceptability (e.g. is it clear and easy to understand, which features they like, their overall satisfaction) of the tool. Data were collected between 21 October 2021 and 30 November 2021. Ethical approval was provided by the Flinders University Human Research Ethics Committee (#2234). All participants gave informed consent before taking part in the survey. This study is reported in line with the Strengthening the Reporting of Observational Studies in Epidemiology (STROBE) Cross-Sectional Studies Statement (Additional file 1).

### Data analysis

Categorical data were analysed using descriptive statistics, whereas the open-ended items were analysed qualitatively. Applying a conventional content analysis approach [22], author SCH initially categorised qualitative responses into groups of responses. Author BK then reviewed the categorisations for any refinements needed, following which SCH and BK finalised the categorisations via consensus, with ALK confirming the final categorisations. The findings for each question were summarised in short textual descriptions.

## Results

**Fig. 1:**
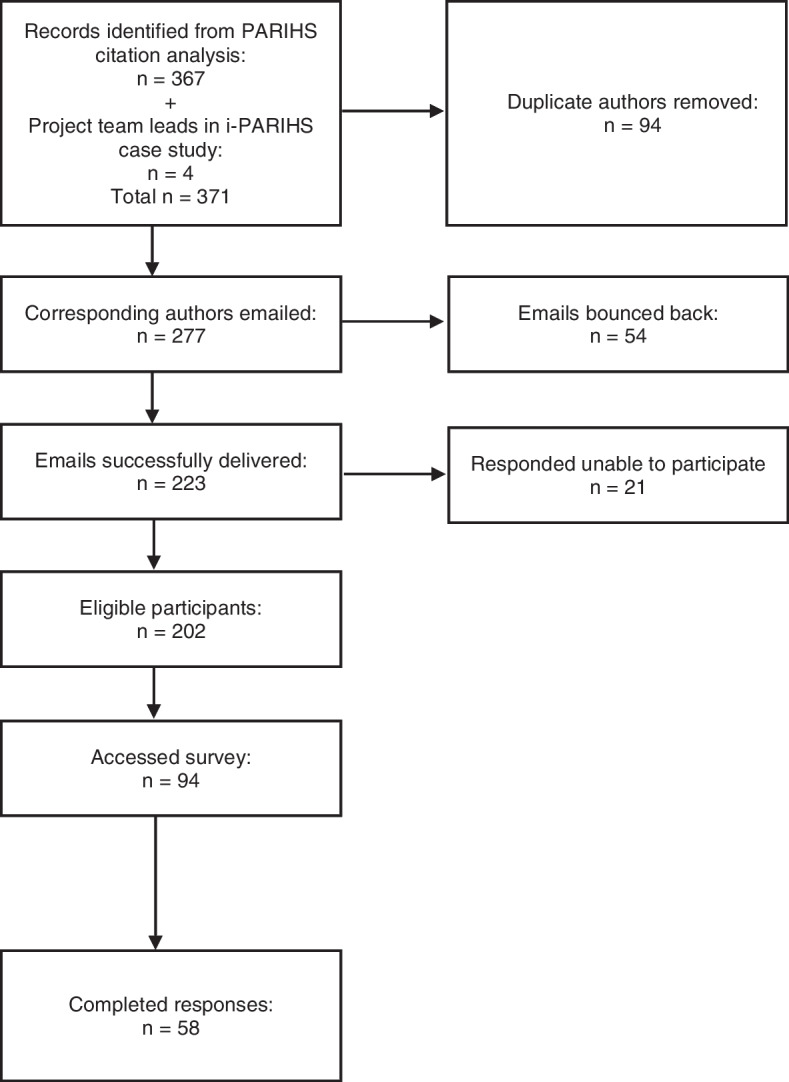
Participant recruitment process

Of the 202 eligible participants, a total of 94 participants accessed the survey and 58 participants returned a completed survey. Figure 1 outlines the recruitment process, and Table 1 outlines the demographics of the participants.

**Table 1:** Demographics of participants (*n* = 58)

**Attributes**		**Number**	**Per cent**
**Current role**	Academic—health professional	7	12.07%
	Academic—researcher	34	58.62%
	Academic—teaching	3	5.17%
	Management/administration	6	10.34%
	Others^a^	4	6.90%
	PhD/doctoral student	2	3.45%
	Project manager	1	1.72%
	Research officer	1	1.72%
**Years in current role**	0–1 year	3	5.17%
	1–2 years	1	1.72%
	2–5 years	9	15.52%
	5–10 years	14	24.14%
	10–15 years	8	13.79%
	15–20 years	8	13.79%
	20 + years	15	25.86%
**Completed PhD**	No	6	10.34%
	Yes	52	89.66%
**Years post-PhD**	0–1 year	2	3.85%
	1–2 years	1	1.92%
	2–5 years	5	9.62%
	5–10 years	13	25.00%
	10–15 years	12	19.23%
	15–20 years	7	13.46%
	20 + years	14	26.92%
**Country**	Australia	5	8.62%
	Canada	10	17.24%
	Ireland	1	1.72%
	Northern Ireland	1	1.72%
	Norway	1	1.72%
	Saudi Arabia	1	1.72%
	Sweden	11	18.97%
	Switzerland	1	1.72%
	Turkey	1	1.72%
	UK	3	5.17%
	USA	18	31.03%
	Not reported	5	8.62%

^a^Clinician-researcher in health service; senior research officer; academic—teaching and research

The findings below report on the participants’ feedback on the ‘Mi-PARIHS Facilitation Planning Tool’ (henceforth, Mi-PARIHS Tool). The findings are structured by (1) tools that participants with experience using i-PARIHS have previously developed themselves, (2) feedback on the background information included with the Mi-PARIHS Tool, and (3) feedback on the Mi-PARIHS Tool’s main content (clarity, use, satisfaction, and improvements).

Of the 58 participants who completed the survey, 38 had previously used the i-PARIHS framework. Table 2 outlines how these 38 participants used i-PARIHS.

**Table 2:** Descriptive statistics on the use of i-PARIHS (*n* = 38)

**Questions**		**Number**	**Per cent**
**How many times have you used i-PARIHS?**	Once	5	13.16%
	1–2 times	7	18.42%
	2–3 times	11	28.95%
	3–5 times	6	15.79%
	More than 5 times	8	21.05%
	Unsure	1	2.63%
**What have you used i-PARIHS for?**^a^	Planning implementation	28	62.22%
	Guiding implementation	26	57.78%
	Evaluating implementation	24	53.33%
**In what settings have you used i-PARIHS?**^a^	Acute	20	37.74%
	Community	10	18.87%
	Emergency	4	7.55%
	Palliative	1	1.89%
	Primary	10	18.87%
	Residential	6	11.32%
	Respite	0	0.00%
	Others	5	9.43%
	Outpatient	6	11.32%
**Do you find i-PARIHS easy to use?**	Definitely yes	13	34.21%
	Probably yes	19	50.00%
	Unsure	2	5.26%
	Probably not	4	10.53%
	Definitely not	0	0.00%
**Would you prefer if i-PARIHS was accompanied with resources/tools?**	Definitely yes	25	65.79%
	Probably yes	9	23.68%
	Unsure	4	10.53%
	Probably not	0	0.00%
	Definitely not	0	0.00%
**Did you develop your own products/tools/resources to assist you in using i-PARIHS?**	Yes	31	81.58%
	No	6	15.79%
	Unsure	1	2.63%

^a^Participants could select all that applied

### Previous tools created by participants

Of the 38 participants who had previously used the i-PARIHS framework, 31 developed tools to help them in their use of the i-PARIHS framework. When asked what types of tools they created, participants listed the following main responses: the development of (1) interview guides and context assessments, (2) facilitation or implementation plans, and (3) data analysis and evaluation plans.

#### Interview guides and context assessments

Specifically, 16 participants listed the development of interview guides informed by the framework. Thirteen participants listed the development of context assessments informed by the framework. Some participants provided additional detail. Specifically, one participant outlined developing a context assessment that graded contextual elements to determine the barriers and enablers. Another participant developed a context survey to elicit perceived gaps and barriers from the staff as well as an additional barrier assessment to determine what needs to be addressed/achieved. Another participant outlined the development of a survey that explained the contextual factors and asked the staff questions about what they perceived would positively or negatively support implementation. One participant outlined the development of a readiness to change survey.

#### Facilitation or implementation plans

Twelve participants listed the development of facilitation or implementation plans informed by the framework. In addition, one participant outlined the development of a facilitation diary, facilitation guide, and teaching materials to train facilitators. Another participant outlined the development of a facilitation tracking guide and a facilitation manual. Another participant also outlined the development of an adapted and more practical guide for facilitators. One participant also mentioned the development of training materials and a problems and solutions log.

#### Analysis and evaluation plans

Regarding analysis and evaluation, one participant developed a process evaluation based on the i-PARIHS framework, and three developed data analysis and evaluation plans informed by the framework. Specifically, 13 participants noted the development of data codes or coding frameworks based on the framework.

#### Other tools created

Outside of the above groupings, one participant mentioned the development of a survey (purpose not described), another participant mentioned the development of a workshop and website, and another mentioned the use of the framework in grant writing.

### Background information

Fifty-one participants evaluated the Mi-PARIHS Tool, a summary of participant responses can be seen in Table 3.

**Table 3:** Descriptive statistics on Mi-PARIHS Tool (*n* = 51^a^)

**Questions**		**Number**	**Per cent**
**Do you find the background content clear and easy to understand?**	Extremely clear	11	21.57%
	Very clear	33	64.71%
	Moderately clear	7	13.73%
	Slightly clear	0	0.00%
	Not clear at all	0	0.00%
**Is there any additional information that should be included?**	Yes	16	31.37%
	Unsure	12	23.53%
	No	23	45.10%
**Do you find this tool clear and easy to understand?**	Extremely clear	6	11.76%
	Very clear	24	47.06%
	Moderately clear	16	31.37%
	Slightly clear	3	5.88%
	Not clear at all	0	0.00%
**How user-friendly do you find this tool?**^a^	Extremely user friendly	6	11.76%
	Very user friendly	24	47.06%
	Moderately user friendly	16	31.37%
	Slightly user friendly	2	3.92%
	Not user friendly at all	1	1.97%
**Please select which features you like about this tool**^b^	Inclusion of instructions	25	14.88%
	This is a ‘living document’ that can be tweaked or adapted to suit a user’s project	31	18.45%
	Domain-specific questions that can be assessed on a scale of barrier to enabler	39	23.21%
	The auto-generated radar diagram/visual representation	36	21.43%
	The barriers are highlighted and populated on a separate page	32	19.05%
**Is this a tool you would use?**	Strongly agree	21	42.86%
	Somewhat agree	20	40.82%
	Neither agree nor disagree	6	12.24%
	Somewhat disagree	2	4.08%
	Strongly disagree	0	0.00%
**At which stage of implementation would you use this tool?**^b^	Planning implementation	30	27.78%
	Guiding implementation	32	29.63%
	Evaluating implementation	46	42.59%
**Overall, how satisfied, or dissatisfied are you with this tool?**	Extremely satisfied	12	24.49%
	Somewhat satisfied	26	53.06%
	Neither satisfied nor dissatisfied	9	18.37%
	Somewhat dissatisfied	2	4.08%
	Extremely dissatisfied	0	0.00%

^a^Seven participants did not complete the tool section

^b^Participants could select all that applied

 Fourty-four participants reported that the background information provided with the tool was extremely or very clear and easy to understand. Seven found it only moderately clear, and no one found it slightly clear or not clear at all. However, 16 did respond that additional information is needed. When asked what additional information they think should be included, participants listed the following main responses: the need for (1) more explanation of the i-PARIHS framework, (2) explanation of the Facilitation Toolkit, and (3) practical support and development of the Mi-PARIHS Tool.

#### More explanation of the i-PARIHS framework

Four participants outlined the need for more explanation and definition of the i-PARIHS framework and the constructs of innovation, context, recipients, and facilitation. One mentioned defining pre-implementation from an i-PARIHS perspective, and another mentioned the need for an explanation on how i-PARIHS differs from other implementation or quality improvement approaches. Furthermore, six participants specifically outlined the need for more definitions and examples of facilitation (e.g. the skills and knowledge requirements, internal versus external facilitators) and information on successful strategies and types of behaviour change.

#### Explanation of the Facilitation Toolkit

Three participants outlined uncertainty around the Facilitation Toolkit and how it relates to or differs from the Mi-PARIHS Tool. Two participants outlined the need for more explanation on why mobilisation of i-PARIHS is required and an explanation on the progression from PARIHS to i-PARIHS and now Mi-PARIHS.

#### Practical support and development of the Mi-PARIHS Tool

One participant outlined the need for more practical information and one other suggested some accompanying graphics or illustrations. One participant suggested the development of case studies that have applied the Mi-PARIHS Tool.

#### Other desired information

Outside of the above groupings, one participant mentioned more explanation on a team approach, another mentioned unpacking what is meant by ‘there is no correct way to use the tool’, and one mentioned clarifying that the three questions at the end are what users should be considering when using the tool.

### Mi-PARIHS Facilitation Planning Tool

#### Clarity of Mi-PARIHS Tool

Of the 51 participants who evaluated the Mi-PARIHS Tool, 30 reported that the tool was extremely or very clear and easy to understand. Nineteen found it only moderately or slightly clear, and no one found it not clear at all. When asked what was not clear, 17 participants responded and listed the following things: (1) rating system and (2) repetition, academic language, and subjectivity of the questions.

##### Rating system

Eight participants outlined confusion with the rating system, noting that some questions are worded in a ‘yes’ or ‘no’ format but the scoring system is a Likert scale. One participant suggested ‘to populate the drop-down menus with short answers as well as numbers, e.g. for the question “Is [insert innovation] informed by strong evidence?”, the drop-down menu might read − 2, very little evidence; − 1, evidence exists but it is inconclusive; 0, evidence is weak; 1, there is moderately good evidence; and 2, there is strong evidence’. Another participant suggested an explanation of the scoring system and the difference between degrees 1 and 2.

##### Repetition, academic language, and subjectivity of the questions

Three participants queried where the questions came from and repetition across the questions, especially in the individual and team recipient questions. Two participants outlined that the wording might be too difficult for those not familiar with the framework and implementation research or those not PhD qualified. Two participants queried how questions can be answered correctly or accurately as they are subjective.

##### Other unclear items

Outside of the above groupings, one participant suggested having a worksheet for the − 1 barriers as well (currently, the worksheet reflects − 2 barriers), and one outlined that the technology/screens were difficult to navigate.

#### Use of Mi-PARIHS Tool

Of the 51 participants who evaluated the Mi-PARIHS Tool, 41 reported that this is a tool they would use, 6 neither agreed or disagreed that they would use it, only 2 somewhat disagreed, and no one strongly disagreed that they would use it. When asked why they would use it, 43 participants responded and listed the following reasons: (1) simple and structured, (2) helpful for assessing implementation, (3) helpful for planning implementation, (4) usable across multiple implementation phases, and (5) aids in communication.

##### Simple and structured

Eleven participants outlined they would use the tool due to it being simple and structured. Of these participants, one detailed that the tool provides a logical approach to a complex problem, whilst two outlined how the tool would help keep efforts focused. Four participants outlined that the tool is simple and easy to use.

##### Helpful for assessing implementation

Seventeen participants outlined they would use the tool due to its capacity to undertake diagnostic and monitoring assessments of the i-PARIHS constructs. Of these participants, seven detailed that the tool is useful for assessing and identifying implementation barriers and facilitators. Additionally, five participants detailed the tool as being useful to assess all of the domains including context, innovation, recipients, and facilitation. Finally, four participants outlined how this tool allows for assessment which helps with identifying what strategies or approaches are needed.

##### Helpful for planning implementation

Eight participants outlined they would use this tool to help develop their implementation plan. Specifically, two participants outlined how the tool could help facilitators with where they need to start and makes facilitation more accessible.

##### Usable across multiple implementation phases

Four participants outlined they would use this tool across the various phases of implementation, specifying that the tool can be used to assess, develop a plan for and evaluate an implementation effort.

##### Aids in communication

Three participants outlined that they would use the tool to aid in communication amongst team members and stakeholders. Specifically, they felt that the tool can provide a clear and visual representation of complex factors and help in getting everyone on the same page.

#### Satisfaction with Mi-PARIHS Tool

Of the 51 participants who evaluated the Mi-PARIHS Tool, 38 were extremely or somewhat satisfied with the tool. Nine were neither satisfied nor dissatisfied, only 2 were somewhat dissatisfied, and none was extremely dissatisfied. When asked if we should include any additional questions, 16 participants suggested the following additions: (1) more explanation of the questions, (2) updates to the questions, and (3) additional questions.

##### More explanation of the questions

Four participants outlined that the questions were fine, but some more information introducing them or explaining how they can be customised is desirable.

##### Updates to the questions

Five participants suggested tweaking the existing questions, specifically to simplify the wording or collapse some into one question. Three of the participants said some of the questions could be better clarified.

##### Additional questions

Seven participants suggested additional questions that could be included. The suggestions related to including questions about population-specific factors, organisational resources, motivation of recipients, specifying patients as separate from recipients, and questions related specifically to the evaluation of facilitation.

#### Improvements to Mi-PARIHS Tool

The final question in the survey asked participants if they had any final comments regarding the changes or improvements that could be made to the Mi-PARIHS Tool. Of the 51 participants who evaluated the Mi-PARIHS Tool, 46 provided a suggestion. These are related to (1) Excel and interface, (2) assistance with barriers, (3) use over time, and (4) question structure and wording. Table 4 provides a summary of these suggestions made by participants and how these were incorporated into the Mi-PARIHS Tool. Meanwhile, Table 5 provides an overview of the suggestions not incorporated into the final version of the Mi-PARIHS Tool, along with considerations and potential plans for their incorporation in the future.

**Table 4:** Suggestions incorporated into the final version of the tool

**Participant feedback**	**Changes made**
**Background content**	
More explanation and definition of i-PARIHS framework and constructs	More detailed definitions provided
Define pre-implementation from the i-PARIHS perspective	More information outlined on the pre-implementation stage as conceptualised by i-PARIHS
How i-PARIHS differs from other frameworks and approaches	It is now clear that i-PARIHS differs from other frameworks due to its focus on facilitation
More definitions and examples of facilitation	Facilitation is defined in more detail with examples of how it can be operationalised
Clarification of what the Facilitation Toolkit is and how it relates to or differs from the Mi-PARIHS Tool	More detail on how the Mi-PARIHS Tool was developed following on from the Facilitation Toolkit
Explanation of why mobilisation of i-PARIHS is required	More rationale provided for why Mi-PARIHS was developed
Explanation of the progression from PARIHS, i-PARIHS to Mi-PARIHS	The history and process to Mi-PARIHS is now explained
More explanation of a team approach	Information now provided on how to use the tool and i-PARIHS more broadly as a team
What is meant by ‘there is no correct way to use the tool’	More detail that this tool is a guide and the rating system is subjective
Clarifying the three questions at the end	
**Mi-PARIHS Tool**	
Lack of clarity with the rating system	The tool now includes prompts/explanations in the drop-down menu to assist with rating
Repetitive questions across individual and team recipients	Individual and team have been collapsed into one overall ‘recipient’ section
Simplify the wording of questions	All questions have been reworded into simple language
More information explaining the questions	More explanation provided in instructions
Change questions to statements	Questions have been turned into statements
Information on the tool as iterative and being repeated over time	More information on how the tool can be used over time in instructions

**Table 5:** Suggestions not incorporated into the final version of the tool

**Participant feedback**	**Current considerations and potential plans to incorporate in the future**
More information on successful strategies and types of behaviour change	Once Mi-PARIHS has been tested and used, we will provide an update on successful strategies and how these fit with the facilitation
Accompanying graphics and illustrations	Future iterations may include more comprehensive graphics and illustrations
Case studies that have applied the Mi-PARIHS Tool	This could be developed in the future once the Mi-PARIHS Tool has been widely used and case studies become available
Questions are too subjective to be answered correctly	In line with the i-PARIHS framework, there is no ‘correct’ way to answer the questions that will result in a defined outcome; the questions are designed to be subjective
Worksheet for − 1 barriers too	Future iterations may include − 1 barriers too; individual users are still encouraged and able to identify strategies for all barriers and enablers
Technology/screens difficult to navigate	The tool can be saved as a pdf and printed to use as a hard copy; note that this will mean the interactive radar diagram cannot be used
Additional questions	The questions were developed based on the i-PARIHS framework; questions that go beyond the framework would need to be tested and validated to ensure they theoretically align
Different platforms other than Excel	This is beyond the scope of the current tool; until future funding is secured that can support the development on a different platform, the tool will remain in Excel as it is a widely used platform
Clarification on what to do with the barriers	This is beyond the scope of the current tool, but future iterations may include strategies that facilitators can use
Matching barriers with strategies	
Links to other instruments and tools	Use of other tools and instruments would be at the discretion and decision of the team using the Mi-PARIHS Tool to ensure it theoretically aligns with their implementation project

## Discussion

The comprehensive 34-item Mi-PARIHS Facilitation Planning Tool supports i-PARIHS users in their (1) assessment of the i-PARIHS framework’s innovation, context, and recipients constructs; (2) development of a tailored facilitation plan; and (3) repetition over time to evaluate the effectiveness of facilitation strategies. To ensure that the Mi-PARIHS Tool meets the users’ needs, its development was conducted in four steps, aligned to the first four steps of the five-step experience-based co-design toolkit [19].

The current study reported specifically on step 4 and presents the feedback and comments from 58 participants who tested the tool and completed a feedback survey. Based on their feedback, we have incorporated all feasible changes (e.g. clarity of questions, improved instructions for use) into the final version of the tool. Appendix. By introducing the Mi-PARIHS Tool and sharing the steps that we took for its development, this article invites the health service researchers and clinicians to actively use, provide feedback, and generate ideas surrounding the tool to ensure Mi-PARIHS’ continued relevance to the most pressing considerations for implementation science.

One such key consideration for implementation science is implementation fidelity, which this tool has the ability to help monitor and document. Implementation fidelity explains the degree to which an intervention is implemented as intended and the degree of fidelity can significantly influence our ability to interpret implementation success [23]. Fidelity raises an important philosophical consideration to reflect on with a framework like i-PARIHS. i-PARIHS prioritises flexibility and being adaptive and iterative to context, all of which are crucial to working within the complexity of real-world practice. However, this tailoring and managing adaptable components can make fidelity impossible to achieve [23]. It is important to balance the need for being adaptive to context with the need to have fidelity to evaluate implementation success. Therefore, the development of this tool was to provide i-PARIHS users with a systematic and theoretically informed way to plan their facilitation plan in the pre-implementation phase of their project, and to use this tool repeatedly during the implementation phase to monitor their fidelity and document changes.

There was no intention with the development of this tool to tell participants how to ‘correctly’ use the i-PARIHS framework. This tool remains true to the philosophy of the i-PARIHS framework that implementation needs to be responsive to real-world context and that it is the role of facilitators and implementers to manage complexity as it arises. Through the development of this tool, we are attempting to generate a new approach to documenting and managing implementation and facilitation without undermining the significance of managing complexity and remaining flexible in our approaches [5, 24, 25]. Our tool balances being flexible and being built squarely on theory-based i-PARIHS and co-design principles. Especially as many real-world implementation efforts face the need to adapt to complex and dynamic contexts, the tool serves as both a timely example for many in the field, to consider, and an encouragement for them, to also share their own theory- and user-driven approaches to implementation.

To ensure end-users can actively use, provide feedback, and generate ideas surrounding the tool, for co-design step 5, *monitor and maintain the experience*, we have set up the following process that directly stems from the work described in this paper. The Mi-PARIHS Facilitation Planning Tool is now publicly available online for users to access (https://www.flinders.edu.au/caring-futures-institute/Mi-PARIHS-tool). To access the Mi-PARIHS Tool, users are prompted to provide their contact information and complete a few short questions (e.g. in what setting they intend to use the tool, how they want to use it). In addition, we outline to users that we will reach out to them over time as part of our plans to ongoingly collect information about their use and satisfaction with the tool. We anticipate that this information will be used to identify future participants for case studies of tool usage and future refinements of the tool.

This tool has strengths and limitations that are important to note. In terms of strengths, this tool is simple and structured. This tool provides a way to work logically through complex problems and assists with tailoring implementation and facilitation strategies to the context and needs of recipients. The tool also allows for the identification of implementation barriers and facilitators. Importantly, this work presents the development of a tool that assists with comprehensively operationalising the i-PARIHS framework, as opposed to focusing on a single element (i.e. context assessment). However, future research needs to examine the utility of this tool over the lifecycle of an implementation project. Furthermore, it is important to note that the use of purposeful sampling, whilst beneficial in ensuring rich and detailed feedback on the tool, meant that our sample was skewed toward PhD academic researchers. Therefore, further work is required to ensure the relevance of this tool to clinicians.

By creating the Mi-PARIHS website and by making the Mi-PARIHS Facilitation Planning Tool freely available, we want to encourage the i-PARIHS framework users to share their experiences of using the tool and also outline the success or otherwise of their endeavours. This will lead to more consistent sharing of results of interventions which in turn will help us understand the mechanisms of successful implementation. We have also been encouraged to learn about the other tools that teams have developed using i-PARIHS, and in addition to the Facilitation Planning Tool, there may be opportunities to co-create i-PARHIS-informed templates that will standardise interview guides, facilitation guides and/or diaries, and training materials. These were all innovations that respondents reported, and the Mi-PARIHS team will continue to engage the wider implementation science community in refining such tools.

## Conclusions

The comprehensive 34-item Mi-PARIHS Facilitation Planning Tool is a step toward making framework-guided implementation more approachable to a wider range of systems and stakeholders, thereby contributing to the more equitable implementation of evidence-based practices and other innovations. It addresses the challenge of systematically assessing core constructs of the i-PARIHS framework to develop tailored facilitation strategies. The Mi-PARIHS Facilitation Planning Tool is available for use at the website https://www.flinders.edu.au/caring-futures-institute/Mi-PARIHS-tool. Its use could improve implementation success by assisting with framework-informed implementation planning that comprehensively facilitates the translation of evidence into practice.

## Supplementary Information

**Additional file 1.** Strengthening the Reporting of Observational Studies in Epidemiology (STROBE) Cross-Sectional Studies Statement.

## Abbreviations

I-PARIHS: Integrated-Promoting Action on Research Implementation in Health Services
Mi-PARIHS: Mobilising Implementation of i-PARIHS
PARIHS: Promoting Action on Research Implementation in Health Services
